# Supporting your support staff during crises: recommendations for practice leaders to develop a relational workplace

**DOI:** 10.1136/leader-2023-000780

**Published:** 2023-12-20

**Authors:** Francesca Dakin, Tanvi Rai, Sara Paparini, Trisha Greenhalgh

**Affiliations:** 1 Nuffield Department of Primary Care Health Sciences, University of Oxford, Oxford, UK; 2 Wolfson Institute of Population Health, Queen Mary University of London, London, UK

**Keywords:** support, COVID-19, capacity, health system, microsystem

## Abstract

**Background:**

The workload and wellbeing of support staff in general practice has been critically understudied. This includes reception, secretarial and administrative workers who are critical in the daily practice function. Currently, only reception staff are mentioned in the evidence base on general practice working conditions, and all support staff are excluded from studies about the impact of the pandemic on healthcare workers’ work and wellbeing.

**Aim:**

To outline the unique work support staff do, the additional burden it places on them, and how the symphony of crises in 2020–2023 compounded those burdens. Additionally, to provide practical advice for practice leaders on how to support staff wellbeing through developing a relational and psychologically safe working environment.

**Methods:**

These findings are drawn from qualitative research (case studies built through observations, interviews and focus groups) conducted in 2022–2023.

**Results:**

Through theoretically informed analysis, we found that support staff do specialist intersectional guiding work to support patients, other staff, and the practice as a whole. We define this as lay translation, specialist-lay translation, and occupational translation. Under crises, the volume of this work grows, complexifies, and becomes more fragmented. Relational and supportive teams were more able to adapt to these challenges.

**Discussion:**

Support staff should be recognised and enabled to perform these specialised roles. Therefore, we provide a set of recommendations for practice leaders to consider integrating into their own workplaces.

**WHAT IS ALREADY KNOWN ON THIS TOPIC**

- Working conditions and wellbeing of support staff in general practice are understudied. These staff work at the intersections of patient lifeworlds, the practice system, and the wider healthcare system. They do delicate interpretational and translational work to support the smooth running of these junctures. In times of crisis, their location in this network means they face additional burdens.

**WHAT THIS STUDY ADDS**

- Support staff have a unique capacity to enable lay-generalist, lay-specialist and occupational translation for better practice function and more appropriate patient care. Our analysis revealed that a relational approach to the general practice workforce is critical. Guidance on how to build capacity and support for relationality would benefit practice staff and leaders.

**HOW THIS STUDY MIGHT AFFECT RESEARCH, PRACTICE OR POLICY**

- Support staff are central in the proper functioning of the healthcare system and good patient outcomes, and further research is needed to understand their work and wellbeing. In providing recommendations to support better staff wellbeing, we hope that practice leaders will be able to make changes to their own practice policies.

## Introduction

 Workloads in general practice have continued to rise beyond prepandemic levels, following an initial dip during the pandemic’s first few months.^1^,^4^ Patients are returning to practices with more complexity, requiring longer primary care management due to late presentation and increased wait times for secondary care.^3^ This ‘new normal’ of higher workloads arises amidst a growing shortfall between the supply and demand of staff (clinical, administrative, secretarial and reception roles).^1 5 6^ The current situation exacerbates the constraints on funding, workforce and capacity from before the pandemic.^5 7^ This context can make it challenging to maintain workplace conditions that support good staff wellbeing, including appropriate staffing and sufficient psychological safety to support teamwork and speaking up.^8^

National datasets collect valuable metrics (e.g., number of consultations)^2^ but cannot capture more subtle information on what staff do to make an imperfect system run. This hidden work, and the mental, emotional and physical impacts thereof, occurs outside of formal recording systems.^2 9^ Where impacts have been studied, the focus has mostly been on clinicians (general practitioners (GPs), GP trainees and specialist nurses).^310^,^15^ While some studies have looked at the unique role of reception staff in facilitating care delivery,^16 17^ noting their work can be obscured,^18^ none have looked explicitly at how workload during times of crisis impacts their wellbeing. Given the complexity and interconnectedness of the healthcare system,^19 20^ the hidden nature of support work,^17 18^ and government calls to increase general practice workloads,^21^ this is an important gap in the literature.

Support staff in general practices tend to reflect the ethnicities of their local areas. The GP support staff workforce broadly reflects the ethnicity data of the UK population,^22^ with support staff respondents to a 2023 National Health Service (NHS) survey self-describing as White (78.9%), Asian/Asian British (6.8%) and Black/African/Caribbean/Black British (1.8%).^6^ It is a highly gendered workforce (95.2% female),^6^ the rate of pay is low,^23^ and many work part time.^6^ This multiethnic, multicultural and multilingual workforce is key to the proper function of general practice. However, they face multiple and intersecting challenges through their work, due to their unique position at the intersections of patient lifeworlds, the practice system, and the wider healthcare system. This paper highlights the specialised translational work that their intersectionality and positionality enables, as well as the unique burden it places on them, to encourage practice leaders provide them tailored support, as guided by our recommendations.

This paper outlines the unique work these staff do, with a novel focus on their capacity for three types of translational work: *lay*, *specialist-lay* and *occupational*, drawing on the work of Greenhalgh, Robb and Scambler who previously developed the concept of ‘lay translators’ in mediated clinician–patient interactions.^24^ We also highlight how working through the crises of 2020–2023 has affected these staff. This includes the COVID-19 pandemic, rising cost of living and the current NHS capacity crisis. It provides practical advice on how to support their wellbeing, and develop a psychologically safe working environment for a variety of practice leaders: partners, practice managers and team leads. These are founded on the principle of the ‘relational workplace’, a term interpreted from the healthcare improvement literature.^8^ It posits that people working in healthcare systems rely on human action, interaction and relationships to enable the adoption, implementation and long-term sustainability of improvements.^8^ By applying the principle of relationality to the work of support staff in general practice, we extend it to propose that it underpins the function of the healthcare system as a whole, and that a relational approach to leadership and teams is required for good practice function and good patient care.

## Methods

### Study design

This paper draws on data collected in 2022–2023 for a substudy of a wider programme of research in 12 general practices to inform the use of digital technologies for care access and delivery in general practice.^25^ This substudy focuses initially on two longitudinal ethnographic case studies built through observations, interviews and focus groups,^26^,^28^ with subsequent shorter case studies to investigate how working conditions in general practice have developed over the pandemic (2020–2023), and how that has affected the people working there.

### Sample and setting

Two English general practice surgeries, both of which have been pseudonymised, one in a very deprived area of London (Perrymore), and the other in a mixed deprivation area of South East England (Easton). Both serviced mixed patient populations regarding class, ethnicity and linguistic preferences. The support staff at our chosen sites reflect ethnicity and geographic location data from the census.^22^ In Perrymore’s region, this workforce was 45.8% White, 24.7% Asian/Asian British, 9.5% Black/African/Caribbean/Black British and 3.8% other.^6^ In Easton’s region, it was 83.9% White and 2.8% Asian/Asian British.^6^

### Data collection

Longitudinal data were collected by FD from two sites over 8 months. While the wider dataset provided us important context on the breadth and unique nature of support staff work, this paper draws on a specific subset of those data. This includes field notes from 7 weeks of observation in various locations within the practices, interviews with 15 support staff, and a focus group with 6 participants working in clinical and support roles. There was some overlap between focus group members and interviewees (n=2 support staff). The interviews and focus group were audiotaped with consent and transcribed. Along with typed and annotated field notes, these were deidentified and loaded onto NVivo software. The rest of the findings are yet to be published.

### Data analysis

Transcripts were read and re-read by FD to gain familiarity before performing abductive analysis to identify possible themes for closer investigation, which allowed us to theorise with the data, without being confined to a single theory.^29^ Analysis presented here focuses specifically on themes relevant to support staff,^29^ and their intersection with issues of equality, diversity and inclusion.

### Patient and Participant Involvement and Engagement (PPIE)

The sub- study has included two arms of PPIE: a project steering group of various staff in GP practices and local focus groups at each longitudinal site to determine local acceptability and priorities.

### Theoretical framework

Our analysis was guided primarily by Maben *et al*’s framework of ‘workplace conditions that matter’.^8^ This interdisciplinary framework, outlined in table 1, which draws on various academic traditions, has three components: staffing for quality (developed in the healthcare improvement tradition),^30^ psychological safety, teamwork and speaking up (from the organisation and management tradition),^8^ and health and wellbeing at work (from the occupational health tradition).^31 32^ We augmented this framework with the feminist sociological concept of invisible (or hidden) work,^33^ that of ‘lay interpreters’ who translate between ‘lifeworlds’ and ‘systems’,^24 34^ and intersectionality, originally developed by Crenshaw in regards to race and gender,^35^ but later expanded into several other axes of oppression. In research on healthcare systems, this concept has thus far been mainly applied to patient populations.^36 37^

**Table 1:** Theoretical framework for considering the wellbeing of support staff at work

Concept	Definition
Staffing for quality	Maben *et al* have described that the number and skillset of staff is a prerequisite for high-quality and safe care. Based on evidence from nursing literature, getting this right can improve conditions for staff by contributing to improvements in wellbeing, sickness rates, retention and burnout.^8^
Psychological safety, teamwork and speaking up	Psychological safety is when team members feel secure in taking interpersonal risks^38^: it enables speaking up, whereby people are willing to ‘voice their observations, questions and concerns, especially to colleagues above them in a hierarchy.’^8^Teamwork is where people in an organisation work collaboratively to complete interdependent tasks.^8^
Health and wellbeing at work	Staff physical, mental and emotional wellbeing in response to pressurised working conditions.^8^ This can cause burnout: a process of negative trending attitudes and behaviours as personal resources become exhausted.^3 39^
Invisible or hidden work	Work that is conducted by ‘focal actors’ at the fringes of defined ‘job roles’, often considered as adjunct to core tasks. However, in healthcare systems, this obscured work can be essential ‘translational mobilisation’ in sustaining the networks of care organisation and care delivery.^33^
Lifeworld/system and lay translators	Originally developed by Habermas,^34^ and applied to healthcare by Mishler,^40^ depicts two ‘worlds’: the lifeworld comprising the family, household and cultural community; and the ‘system’, comprising the economy and state (including the various institutions and bureaucracies with which people must interact). Miscommunication between patients and healthcare professionals (especially but not exclusively when those individuals are from different cultural or racial groups) occurs when the language of the system is poorly translated into the language of the lifeworld. Staff who share ‘lifeworld’ experience can sometimes bridge this gap and provide support and advocacy for vulnerable groups through ‘lay translation’.^24^ This concept has thus far only been applied to patient and clinician interactions.
Intersectionality	This concept challenges us to think about disadvantage and oppression as a multiaxis interaction of individuals different identities in systems of power, such as their ethnicity, gender,^35^ social class, disability status and role in the workplace. It has been applied to patient-focused healthcare inequality research,^36^ and critiques of descriptive but undertheorized patient-focused research.^37^

## Results

Across the two sites, 29 support staff and 2 practice managers were observed over 4 weeks (18 at Easton, 11 at Perrymore). We include practice manager data because of their central organisational role regarding reception and administrative/secretarial staff. Collected demographic data on support staff interviewees are presented in table 2.

**Table 2:** Demographic data of interviewed support staff

Demographic data	No of interviewees
Job title*	
GP support administrator	3
Reception team lead	2
Patient services coordinator	2
Receptionist	1
Senior coordinator*	1
Patient services manager*	1
Phlebotomist/patient services coordinator	1
GP support supervisor*	1
Clinical assistant (administrative role)	1
Practice manager*	1
General operations manager*	1
Ethnicity†	
White British	11
Asian/Asian British (Indian and Pakistani)	1
White other (Polish)	1
Other Ethnic group (Arabic)	1
Did not say	1
Gender	
Female	14
Male	1
Practice	
Easton	10
Perrymore	5
Total	15

* Denotes a leadership position.

† Of represented Office for National Statistics (ONS) recommended categories for country-specific ethnic group data collection https://www.ons.gov.uk/methodology/classificationsandstandards/measuringequality/ethnicgroupnationalidentityandreligion. Where the interviewee specified further, this is given in brackets.

GP, general practitioner.

All qualitative data presented here either use allocated anonymous identifier codes, or pseudonyms when a practice/person’s name is mentioned in the data. Identifiable comments in quotes have been fictionalised to support deidentification.

### Staffing for quality

#### Workload and staffing

All support staff mentioned their high workload. It was stated during observations and in all interviews that workload had increased after the first 6 months of the pandemic and continued to rise during the data collection period. Causes ranged from more patients returning post-lockdowns, the integration of new digital technologies, vaccination and booster drives, and increased workload from secondary care. This work came alongside increasing patient hostility and frustration from system-wide wait times and remote delivery pathways and more exposure to discrimination based on nationality, linguistic markers and ethnicity.

Much of these new forms of work are ‘invisible’ within roles: undocumented by auditing processes and unseen by patients. It can be completed in times or places outside of the practice’s opening hours or estate: through arriving early, staying late or working unpaid hours at home. This meant that staff who took on this overflow work, often due to their concerns for patients and sense of responsibility for other staff, had less opportunity to unwind from the mental and emotional challenges of their role.

This higher workload meant that learning new skills or training others formally or informally became harder. It afforded few opportunities for team-focused training or other activities that build morale and informal networks for support (such as shared lunchtimes or other breaks). Group learning for task-load sharing had the capacity to help support the wider team during acute staffing problems, as demonstrated by team leads or multirole staff that were able to step in and take on additional workload burdens when necessary.

#### Lay, specialised-lay, and occupational translation

Analysis showed that patient and support staff interaction involved guiding work (lay interpretation) to navigate the patient through the healthcare system and appropriately allocate resources. Some members of the teams had pooled knowledge of how to do this, due to their experience the post or because of wider clinical or administrative experience in other roles. This knowledge was often passed between staff informally during the working day. Receptionists had a particularly intimate knowledge of how the practice system functioned from an access and resource allocation perspective, and knew what information, keywords or contact timing would open doors for patients. Staff shared general tips with patients, such as the best time of day to call for particular needs.

Some support staff offered additional specialised guidance to particular patients due to experiences with particular cultures, languages, social classes, government structures and disabilities. These specialist-lay interpreters were often minorities in the wider team and practice structures, but the number of patients requiring their specialised support was comparatively much larger. Therefore, they could face a disproportionately higher sense of responsibility for such patients. They also faced the problem of educating other members of staff about the differences between some patients’ lifeworlds and theirs. These collusive burdens expose staff to additional abuse from patients, emotional strain and create barriers between colleagues when a conflict in cultural or class identity surfaced.

Less overt friction was also evidenced during observations through microaggressions or comments, and specific moments in interviews. They could occur between support staff, when support staff interacted with the wider practice workforce, or with patients. One practice made active efforts across roles to be more inclusive and address systemic discrimination which helped to facilitate a more open learning environment (though biases could still surface), whereas in the other, prejudiced language and behaviour largely went unchecked.

Occupational translators, who worked across the margins of different teams, were able to smooth frictions in interdependent work by drawing on their knowledge of the particularities of how different teams, infrastructures, or processes worked. Our dataset included occupational translators that spanned the boundaries of administration, reception, and clinical roles, which could directly support better working relationships and better patient care. Supporting data for this section are outlined in table 3.

**Table 3:** Supporting data for ‘staffing for quality’

Subtheme	Supporting quotation
Workload and staffing	‘It’s only me who does e-consults at the moment, we never did them before and now we get 50 through a day. I’m the reception team lead so I need to deal with everything else that comes in, and also them. I was in at seven o’clock this morning, I don’t start until eight. Just so I could clear the backlog from yesterday. I’m having to come in out of my own time because I just don’t like it being there. It just gives me pure anxiety. I’ve got a laptop at home, so sometimes I log on at home, and just do some work, but then I don’t feel like I have a break.’—S2S5 Reception Team Lead Interview
Specialised-lay translation(supporting patients)	‘I heard Tania speaking in Urdu (as she later clarified). She had gone to the front desk to take over checking-in a patient after hearing them speaking Urdu—she is the only person in reception who speaks it. She told me it helps to speak to patients in their preferred language so that she can understand what they need. She later told me she speaks five languages—Urdu, Patwari, Punjabi, English, and some Hindi. This mix is because of the regions she and her partner’s families are from, and where she has grown up. She said these languages help with Indian, Pakistani, and Nepalese patients at the practice—she notices when people on the phone are struggling, or if they come in with an English-speaking family member, and will offer to speak in these languages based on what she’s hearing. She told me the Indian community here is quite small, so you get to know everyone.’—Excerpt from Observations at Easton
Specialised-lay translation(staff burden)	‘There was a(East African/Black)man came in, in his 30s, wanting an emergency appointment. He was screaming and shouting(…)And the man said is it because I’m Black? You’re racist(…)So I went out there and I said, okay, guys, leave, I’m going to have a chat with this guy. Calmed him down. Obviously, the colour of my skin probably calmed him down more than anything. And I try to give him what he wanted, put my clinical system hat on.(…)I did not mention the fact that he called the [White British] receptionist racists, but the receptionist made sure to tell the doctor that he did. So that worked in the(receptionists’)favour. So he did not get an appointment. They were like, you should not be helping him, he called us racist. And I said, that’s not an insult. You know, that’s not a reason to block him from getting care.(…)So I’m getting emotional. He’s getting emotional. So it’s just really difficult. Really, really difficult. That’s just one example when trying to bend the rule for one person, but the ends up kind of like, you know (shrugs).’—S1S7 Clinical Assistant Interview
Occupational translation (supporting patients)	‘I get to know the patients a bit more doing bloods, I know more about them, and enough about the clinical side to know it isn’t our decision in reception. There was a gentleman that I know. And he was saying on the phone to another receptionist 'I don't feel well, I don't know what's wrong'. She said it wasn’t urgent. But he never phones, that was a red flag to me straight away. So I said, quietly 'he needs to go on the urgent list'. She would only put him down for urgent bloods, with me. When he came in I went straight to the nurse and I said, you better come see him. I said his nose is black his legs are swollen. And he's not holding his chest or tummy. He looks dreadful. She done him an ECG. He's only having heart attack. He never had no chest pains. He never had no sweaty, clammy, he never had no left arm. None of what reception are trained to identify. Honestly, he was having a heart attack. I knew there was something wrong because he called. That's why I like them to talk to me in bloods.’ S2S9 Phlebotomist/ Patient Services Co-ordinator Interview

### Psychological safety, teamwork and speaking up

#### Practice unity and its implications

Leadership was crucial to engendering psychological safety in staff across roles. This was important in setting a supportive culture of teamwork. Support roles can have fast turnovers, so ensuring a team-working culture relied on the actions of staff with system authority (partners, practice managers, team leads) or social leaders (long-standing and well-established team members).

In one practice, a non-hierarchical approach supported interteam interaction for both social and clinical purposes. This supported staff to feel that they were a ‘family’, and were more likely to ask for advice, check on and help each other. This behaviour was modelled from the top and embodied by partners, practice managers, and team leads. In a setting where hierarchies were more present and enforced, divisions developed between and within teams.

The presentation of a unified whole-practice team was particularly important for reception staff, as the perceived hierarchy of the practice (where support staff rank lower) informed how patients approached and interacted with them. These perceptions derived from wider social influences such as community, media and government narratives, but could be reinforced or disrupted based on the whole team’s response. Where staff across and between roles spoke positively to patients and each other about the work that support staff do, learnt their roles to provide task support, and ensured consistency of patient messaging from clinical and support staff groups, our data reflected that negative interactions could be avoided or de-escalated, and the impact on staff mitigated.

It should be noted that developing a more unified ‘family’ environment may not entirely prevent people feeling excluded if they are in a sociocultural minority, identify less strongly with the team (due to cultural, linguistic or socioeconomic differences), or do not socialise in the same way (eg, due to neurodiversity). This was particularly true when social leaders actively excluded those people. Often, excluded people found their support networks from their personal lives, and therefore, had no safe formal escalation paths.

#### Social bonds

When a team identity was maintained consistently, it could strengthen social bonds within the team, had the potential to diminish the impact of differences in social class and ethnicity, and could improve relations and recognition of work between teams with external parties (such as patients). Occupational translators, who worked across teams and translated between them, were key in this.

Disruptions occurred at the margins of roles: where teams’ work overlapped, interactions with/between patients, or where the practice interacted with other areas of the healthcare system. Conflict could occur because of non-unified communications to parties outside of the practice workforce, for example, in responses to patients or secondary care. Divisions could also form when the team were not treated equally. Our data reflected this in an array of observed privileges like access to resources for working from home, allowance of sickness days and access to an ergonomic workspace or more reliable technologies. Supporting data for this section are outlined in table 4.

**Table 4:** Supporting data for ‘psychological safety, teamwork and speaking up’

Subtheme	Supporting quotation
Unified practice team	‘They’re really good in reception. If they can hear someone having a difficult time, quite often they’ll step in and say, let me help, because sometimes if a patient hears it from a receptionist and doesn’t like what they hear, they think that, well, they must be lying. But then hears it from *another* receptionist, they’ll take it.(…)sometimes I can go ‘hang on a doctor’s just walked in the room’ and they confirm what I said, and the patient will go ‘oh, right, then.’’—S2S10 Patient Services Manager
Lack of practice unity	‘Sometimes people are angry or upset, especially if they want something that we can’t give them. Particularly if a doctor says to patients, call me anytime, and I’ll call you back, which is lovely for the patient. But it means that if the patient then calls back, and *they* expect to get called back that day within an hour. And when we say ‘no, like they’re completely fully booked.’ In the doctor trying to be there and available for the patients, it actually makes it more difficult for reception to manage, and puts pressure on the doctor.’—S1S10 Patient Services Manager Interview
Social exclusion	‘When I was when I was child, I was diagnosed with Austism,(…)I’ve got dyspraxia as well. So, but it’s like, when you go to work. You have to block all that. And you have—you do a show, you’re on show(…)There’s certain people I don’t relate to in there(…)I just feel like there’s a bit of a clique with certain people. And I’m not in that clique(…)Thing is I think certain people have influence on certain things. When I first started working I almost left(…)I speak to my mum, and then I feel better. So it’s fine.’—S2S2 Patient Services Co-ordinator Interview
Strong social bonds	‘I started three months ago. So when it comes to how the trainings been, it’s been full on. But the surgery itself has been really supportive, not just the receptionists, the doctors, the nurses too, and the managers. And they come and they tell you something new as well(…)the surgery here feels more like a family.(…)If you do something wrong, or you make a mistake. You don’t get told off right, they explain to you, and then they teach you how to do it the right way.’—S1S1 Patient Services Co-ordinator Interview
Inequity among staff	‘There are only two people working in reception today. Orla says ‘It’s a nightmare today, it’s so busy and there’s no staff(…)It’s the same people over and over again going off sick and working from home, and it’s the people in charge who get paid twice as much as us. It ain’t fair.’—Excerpt from Observations at Perrymore

### Staff health and wellbeing at work

#### Burnout and invisibility

The health and wellbeing of reception staff have been seriously challenged by the multiple crises faced over the past few years. Some have felt that more acutely than others, but there is consensus in our data that the pressures those crises have created are not going away soon. The unyielding workload pressure contributes to staff burnout, and feelings of invisibility which go beyond ‘hidden’ everyday work. For many of our participants, nearly all of whom were female, this burden came atop managing the pressures of domestic, caring and familial responsibilities, as well as juggling precarious financial positions, with participants often working multiple part-time roles and navigating state credit systems.

The people themselves are obscured through lack of engagement with peers, lack of acknowledgement from other members of staff and through being physically removed from other staff. Promises of support without clear mechanisms to enable access caused more harm by triggering moral injury through accusations of fraudulent behaviour, destabilising staff’s willingness to continue to work beyond their capacity.

#### Impact of prejudice

Some staff faced particular challenges to their mental health because of the very same cultural and linguistic markers that, in other situations, give them the tools to act as specialist-lay translators. Staff can often be caught at the intersections of systems of power, such as those of the practice staff hierarchy, social class and language. These experiences placed additional emotional burdens on practice staff whose identities within and outside of the practice put them at a disadvantage in those systems. Supporting data for this section are outlined in table 5.

**Table 5:** Supporting data for ‘staff health and wellbeing at work’

Subtheme	Supporting quotation
Burnout	‘Yeah, there are days I’ll admit that I go home and, and I just cry(…)I’m just like really trying to help them. So now patients are like, ‘well I want everything *now*’ when you know, unfortunately, they can’t have everything now. Expectation is high. There’s a lot of frustration as well. And I feel their frustration. But I think they feel that because a lot of them didn’t come during COVID with the media saying that ‘oh, well, GP isn’t there’. We were here. We were working, we were here.’—S2S4 Senior Patient Services Coordinator Interview
Invisibility and inaccessible support	‘I felt like I was fighting this kind of invisible wall, it’s like fighting to be heard and screaming so loudly, but no one’s really kind of listening to you. They just kind of like, oh, yeah, they’re fine upstairs. No, we’re actually really struggling(…)After accessing NHS staff therapy)I got a note to file from HR to say, you know, this is kind of fraudulent behaviour. You were accessing therapy during work hours. I didn’t hide anything. There’s literally emails circulating everywhere, posters everywhere, encouraging you to access therapy, but somehow they didn’t link the fact that we were doing therapy during work hours, because where else would I do it? I’m at work 24/7.’—S1S7 Clinical Assistant Interview
Impact of facing prejudice	‘The lady had dementia so she wanted me to speak with her husband, her husband was very rude person. Very. So it was very unpleasant talk, it was pretty bad(…)he wanted to speak with one of my colleagues. He said that he wants to speak with someone ‘professional’(…) on the phone with the team lead he said that she’s supposed to pick better members of her staff, because he didn’t understand my English ‘She was talking to us like a total idiot’.(…)So it’s taken me a while to just get the confidence again in myself to be able to call the people and ask them to book their appointments(…)I was thinking he’s probably right, I cannot speak English good enough.’—S2S8 GP Support Administrator Interview

## Discussion

### Summary of key findings

Our findings highlight that developing a relational workplace can facilitate better working conditions for support staff, and better staff wellbeing. We highlight the unique translational skills of these staff, developed through operating at the intersection of a bureaucratic system and complex personal lives. Such specialised work is important in facilitating patient access and good practice function. When viewed through the lens of intersectionality, we see that some staff can provide specialised translation to patients from shared communities, or communities with common linguistic or cultural traits. This helped us to refine the concept of lay translators as applied to support staff into two subcategories: generalist-lay and specialist-lay, to reflect the generalised and community-specific translational work that we observed. We also include an adjacent category of occupation translators: individuals who work across occupational groups in dual roles, using their knowledge of each to smooth communication and improve working practices.

Despite their unique skills, our data show these staff are also more likely to face abuse from patients and other staff due to intersections of class, ethnicity, language, and perceptions of practice hierarchy. We also found that support staff are more likely to be female, less well-paid, part time, living in deprived areas, and managing domestic pressures from their personal lives.

The work and needs of support staff are often hidden, made invisible, or deprioritised, and can be obscured by the hierarchical nature of GP practices and the public perception thereof. To guide practice leaders we have developed recommendations to support the recursive work of developing and maintaining a relational workplace, in which support staff, and their value, may be better ‘seen’. These recommendations are not yet user-tested, and as such we encourage practice leaders to engage with their staff directly to determine their local applicability. This is presented in table 6, structured using our previously discussed theoretical framework.

**Table 6:** Recommendations for practice leaders to better support ‘support staff’, as guided by our theoretical framework displayed in table 1

Concept	Recommendations for leaders
Staffing for quality	Ensure your no of full-time or part-time support staff is adequate for your practice’s requirements. Ensure staff have time to be trained in all tasks in case of staff shortages. Shift from a demand-led service to a capacity-led service. Team leads should regularly check on the fairness of workload distribution. Encourage informal skill development and intrateam support by expanding social opportunities for example, with group lunchtimes, break times and training. Be aware of the unequal burden faced by team members from underserved communities: ensure they are well supported and have a safe route for escalating disproportionate workloads.
Psychological safety, teamwork and speaking up	Model the behaviour you want to see in your practice: the conditions for psychological safety, teamwork and speaking up begin at the top and must be consistently reinforced at multiple levels (including formal and social leaders). Establish pathways for escalating staff issues safely and privately, so that all staff have a support route should they need it. Recognise that some people may not participate in the practice’s social network because of their own needs, caring responsibilities, backgrounds, or preferences. Care should be taken not to reinforce exclusion of such staff. Present a united team front to support better interteam and intrateam interactions. Be clear and consistent around managing patient expectations across teams: lack of clarity around what the standard care offer is can make equitable management of patients requests difficult. Develop or share multilingual patient education resources about your particular practice’s function to remove some of the translational burden from support staff.
Staff health and wellbeing at work	Ensure knowledge about mental health support services are shared and access is consistently supported. Develop peer-support systems. Ensure provision of resources is appropriate to the role, and be clear about why some roles might have access to different resources. Respond swiftly and appropriately when staff face abuse or discrimination from patients.
Lifeworld/system and lay translators	Know your staff, and know your patients: identify your generalist-lay and specialist-lay translators and their particular community (or lifeworld). Support and recognise their translational work. In mapping your lay translators, identify where you are missing bridges to communities that your practice serves. Think about how you can provide targeted support for them. Identify the occupational translators: those who work at the intersection of occupational groups, and therefore, have unique situated knowledge. Support and recognise their translational work. Ensure the practice does not take advantage of the unique skills that lay, specialist and occupational translators offer. Regularly check in to ensure that they feel their workload is fair and appropriately acknowledged/remunerated.

### Comparison with previous literature

This paper extends the existing literature on support staff, which has thus far focused on the work of receptionist staff,^16^,^18^ to include administrators and secretarial workers. It does this with a further focus on their working conditions and the impact on their wellbeing amidst the multiple crises of 2020–2023. As such, this paper makes a unique contribution in documenting the experiences of these staff, and suggests how practice leaders can better support them. It also expands Maben *et al*’s structure of ‘working conditions that matter’^8^ to general practice, with a focus on support staff. Our analysis has also extended the concept of ‘lay translators’^24^ by applying it to support staff for the first time and using an intersectional lens to reveal three types of translational work done by support staff: lay, specialist-lay and occupational.

### Implications for leadership

Developing a relational workplace strengthens the human infrastructure of the primary care system. A secure and psychologically safe workforce facilitates openness to adaptive and innovative delivery of care,^8^ which can help to meet the unique needs of the variety of patients served by general practices across the UK.

### Strengths and limitations of this study

This study aimed at depth of engagement over a breadth of study sites so as to support theoretical development. Interpretation of our recommendations needs to take into consideration the heterogeneity of practices across the UK, as well as their workforce and patient profiles. This paper does not represent the full dataset of the study, as the aim of the paper was to highlight findings relating to support staff.

### Suggestions for further research

There are other hidden workforces beyond those outlined here whose work is critical to the functioning of the NHS, which we encourage future research to engage with. In the case of general practice support staff, more work is needed to uncover the complexities of their hidden and invisible work, and to understand how to support and reinforce this critical component of the primary care workforce.

## Data Availability

All data relevant to the study are included in the article or uploaded as online supplemental information.
